# Preschool Teachers’ Attitudes about Inclusive Education and Its Influencing Factors in China

**DOI:** 10.3390/bs14100904

**Published:** 2024-10-07

**Authors:** Xiaomei Peng, Toby Long, Xueyun Su

**Affiliations:** 1 Department of Early Childhood Education, Faculty of Education, East China Normal University, Shanghai 200062, China; pengxiaomei027@126.com; 2 Center for Child and Human Development, Georgetown University, Washington, DC 20007, USA; longt@georgetown.edu; 3 Department of Early Childhood Education, Faculty of Education & Changning Maternity and Infant Health Hospital, East China Normal University, Shanghai 200062, China

**Keywords:** inclusive education, attitude, preschool

## Abstract

Background/Objectives: The purpose of this study is to describe the current status of preschool teachers’ attitudes about inclusive education and discuss the factors that influence these attitudes. Methods: A total of 449 preschool teachers who have students with disabilities or special educational needs in their classrooms and 638 teachers without students with disabilities or special educational needs in their classrooms volunteered to complete the online survey. The survey included two components: a Basic Information Questionnaire and the Early Childhood Inclusive Education Attitude Questionnaire (ECIEAQ). Data were analyzed using descriptive statistics, *t*-tests, and ANOVA. Results: Scores in the two dimensions of Positivity and Promotion were higher than those in the two dimensions of Resistance and Passivity, indicating an overall positive attitude about inclusive education. Attitudes about inclusive education significantly differed by gender, preschool location, inclusive education training opportunities, and receipt of a special education financial allowance. Inclusive class teachers who are female, aged over 30, teach classes with a child-to-teacher ratio of more than 14, and who work in urban areas show higher levels of Promotion or Resistance than general class teachers. Conclusions: Overall, preschool teachers hold a positive attitude about inclusive education. Training opportunities and a special education financial allowance should be provided to foster positive attitudes. Certain groups of inclusive class teachers may need more support and resources to implement inclusive education.

## 1. Introduction

Inclusive education in China occurs in a different context than Western inclusive education. Initially the goal was to increase the enrollment rate of children with disabilities in compulsory education, as the enrollment rate of children with disabilities in China has exceeded 95% [1], there has been a shift from quantity to a focus on quality, for which, the teacher is a very critical element. The Division for Early Childhood (DEC) and the National Association for the Education of Young Children (NAEYC) have proposed three features of high-quality preschool inclusive education, which are access, participation, and support [2]. Teachers’ attitudes about inclusive education (IE) are crucial to implementing inclusion successfully. Attitude is a significant predictor of willingness to teach inclusive classes and the intention to use special education techniques which can affect the achievement of all children [3,4,5]. Understanding teachers’ IE attitudes is the precursor to enhancing teachers’ inclusive behaviors [6].

It is commonly recognized that inclusion is a process that helps overcome barriers limiting the presence, participation, and achievement of learners [7]. The term “Learning in Regular Classroom” (LRC) was developed in the 1980s in China in response to the global trend of IE. LRC was promoted by the Central Government of China to include students with disabilities in China’s Nine-year Compulsory Education program (6 years of elementary school and 3 years of junior high school). LRC contributed to a major increase in the enrollment of students with disabilities [8,9]. In early childhood education, a similar term, “Care and Education in Regular Preschool” (CERP), was developed. The term CERP was used in special education promotion policy documentation but not in legislation. In 2020, China’s first Preschool Education Law stipulated (Article 23) that it is the responsibility of the people’s government to promote inclusive education in the region and that preschools shall accept children with disabilities [10]. In this study, the term inclusive education is in accordance with the Chinese context, which means providing learning opportunities for children with disabilities or special educational needs in regular schools.

As cited by Ahmmed (2012), success in implementing effective IE is contingent on teachers’ positive attitudes towards IE [11]. IE policy initiatives increase students’ access to education [12], but the enactment of policy at implementation levels requires exploration [13]. In particular, it is important to determine the similarities and differences between the attitudes and influencing factors of inclusive class teachers (ICTs), who have taken the lead in implementing IE, and the attitudes and influencing factors of general class teachers (GCTs), who are now welcoming children with disabilities in their class after the new legislation in China. This information can also inform teacher professional development programs.

Teachers’ attitude about IE can be described as generally positive and as improving overtime [6,14,15]. Previous research shows that possible influencing factors of teachers’ IE attitudes include gender, age, level of education, IE training experience, rural context, teaching position (special education teacher or general class teacher), years of teaching, being or having been an inclusive class teacher, and school support [3,6,11,15,16,17].

However, few studies compare IE attitudes of teachers with different positions (ICTs and GCTs) and explore the interaction of demographics and teachers’ positions in IE. The aim of this study is to understand teachers’ IE attitudes and their influencing factors. The following two research questions are addressed:

1. What are the current status and influencing factors of preschool teachers’ inclusive education attitudes?
2. What factors interact with teachers’ positions to impact teachers’ IE attitudes?

## 2. Materials and Methods

### 2.1. Participants

A total of 1087 preschool teachers participated in the online survey, 449 ICTs who have children with disabilities or special educational needs (SEN) in their classroom and 638 GCTs who do not have children with disabilities or SEN in their own class. In total, 14.1% of the participants are from Beijing (*n* = 154), 9.9% from Shanxi (*n* = 108), 39.2% from Henan (*n* = 426), and 36.6% from Shandong (*n* = 398), while 0.1% had missing location information (*n* = 1).

In total, 95.5% of ICTs and 97.8% of GCTs are female, which is in line with the national data on the female preschool teacher percentage, which is 97.78% [18]. Participants have a variety of academic qualifications and majors, as shown in Table 1. A total of 2.5% of all participants work in rural preschools. In total, 74.8% are from model preschools, 11.6% are from Level-1 preschools, 9.8% are from Level-2 preschools, and 3.8% are from non-rated preschools (the preschool level is given by the government after assessment regarding preschool ideology, care and safety, the education process, environment creation, and professionals. The levels ranking from high to low are model preschool, Level-1 preschool, Level-2 preschool, and Level-3 preschool).

### 2.2. Instrumentation

An online survey, consisting of 2 components, the Basic Information Questionnaire and the Early Childhood Inclusive Education Attitude Questionnaire (ECIEAQ), was developed, posted online, and distributed through We-Chat groups. Teachers participated on a voluntary basis. The survey began with a welcome message that explained the purpose of the study and informed participants of their right of confidentiality, and the right to withdraw at any time. An informed consent item at the beginning of survey was checked by all participants. Also, the survey did not include personal information that could identify a teacher.

The study was approved by the Institutional Review Board of East China Normal University (IRB number HR 510-2020).

#### 2.2.1. Basic Information Questionnaire

Demographic information was recorded, including gender, age, academic qualification, major, location of preschool (urban or rural), number of children and teachers in the class, monthly salary, whether the teacher has children with disabilities or SEN in the class, whether a special education financial allowance is received, and the number of extra working hours completed weekly. Since the concept of SEN is not commonly known in preschools in China, it was noted in the survey that children with SEN are children who are developmentally delayed or have special educational needs in certain areas such as language, cognition, motor, or social–emotional areas compared to typically developing children.

#### 2.2.2. Early Childhood Inclusive Education Attitude Questionnaire (ECIEAQ)

An attitude survey was developed based on Leyser and Kirk’s (2004) ‘Attitude Toward Inclusion/Mainstreaming’ Scale as well as Wen’s (2010) ‘Attitude Toward Inclusive Education’ Scale [19]. The original questionnaire by Su et al. comprises 18 items, targeting teachers’ attitudes towards children with Autism Spectrum Disorder (ASD). The item phraseology was modified to be more universal and less specific to children with ASD, i.e., changing “children with ASD” to “children with disabilities and special educational needs”.

The ECIEAQ is an 18 item, five-point Likert scale, with four dimensions. Example items are “Inclusive education is an important means of achieving equality in education” and “Inclusive education contributes to the development of ordinary children’s social skills and better prepares them for entering society”. The Positivity dimension includes the recognition of the right to education of children with disabilities or SEN and the value of IE. The Resistance dimension includes the perception of adverse effects and the inconvenience and practical difficulties of IE. The Promotion dimension includes the acknowledgement that IE should be promoted and willingness to take children with disabilities or SEN in their own class. The Passivity dimension includes the belief in segregated education systems and the belief that there are limited things teachers can contribute in IE.

### 2.3. Data Analysis

A total of 1138 teachers participated in the online survey. Answers for each item were reviewed manually, and vacant questionnaires and obviously unreasonable answers (the number of extra working hours completed weekly being over 100, more than 10 teachers in one class, etc.) were deleted from the analysis. A total of 1087 were found to be usable completed questionnaires for analysis. Statistical Package for Social Sciences (SPSS) version 22.0 was used for data analysis.

## 3. Results

### 3.1. Factor Analysis Result of ECIEAQ

The Kaiser–Meyer–Olkin (KMO) value (0.925) and Bartlett’s test of sphericity (χ^2^ = 21,716.755, *p* < 0.001) indicated that the data were suitable for factor analysis. The interpretation rate of cumulative variance was 82.875%. Results of the Exploratory Factor Analysis (EFA) yielded four factors: Positivity (items 1~8), Resistance (items 9~11), Promotion (items 12~15), and Passivity (items 16~18). Cronbach’s alpha for each factor and the whole scale was 0.871–0.969, indicating high internal consistency. After model modification, the Confirmatory Factor Analysis result showed the factor loading was 0.702–0.950; Comparative Fit Index (CFI) = 0.927; Tucker–Lewis index (TLI) = 0.911. Item 12 was excluded for low factor loading (0.653).

### 3.2. Results of ICTs’ Inclusive Education Attitudes and Variation across Demographic Groups

To compare teachers’ attitudes in different dimensions, ANOVA analysis was conducted. As shown in Table 2, significant differences between teachers’ attitudes in different dimensions existed in both ICTs and GCTs. The results of the Least Significant Difference (LSD) analysis showed that the ranking of dimensions from the lowest to the highest are Resistance, Passivity, Promotion, Positivity.

To investigate the variance in ICTs’ IE attitudes across different demographic groups, independent sample t analysis was conducted on gender, highest academic qualification, major, location of preschool (urban or rural), level of preschool, whether the teacher participated in IE training, and whether a special education financial allowance is received. The results indicate that four factors have an impact on teachers’ IE attitudes, gender, location of preschool, IE training experience, and special education financial allowance, as shown in Table 3.

Compared to female teachers, male teachers had significantly higher scores in the Positivity dimension (*t* = 2.858, *p* < 0.01). No difference was found in the other three dimensions.

Compared to teachers from rural areas, teachers from urban areas had significantly higher scores in the Positivity dimension (*t* = 7.792, *p* < 0.001) and Promotion dimension (*t* = 4.163, *p* < 0.05). No difference was found in the Resistance and Passivity dimensions.

Teachers who participated in IE training had significantly higher scores in the Positivity dimension (*t* = 18.206, *p* < 0.001) and Promotion dimension (*t* = 26.787, *p* < 0.001) than teachers who had not participated in any training.

Teachers who received special education financial allowances had significantly higher scores in the Positivity dimension (*t* = 7.103, *p* < 0.001) and Promotion dimension (*t* = 7.262, *p* < 0.001) and significantly lower scores in the Resistance dimension (*t* = 9.164, *p* < 0.001) and Passivity dimension (*t* = 4.907, *p* < 0.001).

### 3.3. Comparison of ICTs and GCTs: Subgroups Where Differences Exist

Considering the full sample of the 1087 participants together, ICTs had significantly higher scores in Resistance (*t* = 5.710, *p* < 0.005) and Promotion (*t* = 12.901, *p* < 0.001) than GCTs. There was no significant difference in the scores of the Positivity and Passivity dimensions. After further examination, gender, age, and class size had an impact on the difference in attitude between two groups, as shown in Table 4.

Female ICTs scored higher in the Resistance (*t* = 2.277, *p* < 0.001) and Promotion (*t* = 3.412, *p* < 0.001) dimensions than female GCTs. For male teachers, there was no difference in all dimensions.

For teachers aged 30 and below, ICTs scored higher in the Promotion dimension (*t* = 14.077, *p* < 0.001) than GCTs. For teachers aged 31 and above, ICTs scored higher in the Resistance dimension (*t* = 4.264, *p* < 0.05) than GCTs.

For teachers in classes with a child-to-teacher ratio of 14 and less, ICTs scored higher in the Promotion dimension (*t* = 5.557, *p* < 0.05) than GCTs. For teachers in classes with a child-to-teacher ratio of more than 14, ICTs scored higher in the Resistance dimension (*t* = 4.237, *p* < 0.05) and Promotion dimension (*t* = −6.944, *p* < 0.01) than GCTs.

For teachers working in urban preschools, ICTs scored higher in the Resistance and Promotion dimension than GCTs. This pattern was not found in teachers working in rural preschools.

## 4. Discussion

### 4.1. Current Status of Teachers’ IE Attitudes

Consistent with previous research, teachers’ attitudes about IE are generally positive [6,14,15], for both ICTs who currently teach children with disabilities or SEN and GCTs who do not.

Teachers’ attitudes about IE are more nuanced than the dichotomy of positive and negative. Unlike the three-factor structure (affective, behavioral, and cognitive) of preschool teachers’ IE attitudes found by Lohmann et al. (2016) using the Multidimensional Attitudes toward Preschool Inclusive Education Scale (MATPIES) [20], Lu et al. (2023) found a four-factor structure (affective, behavioral, cognitive—arrangement, and cognitive—significance) through a Chinese sample, indicating nuances in attitudes towards IE [21]. Similarly, as shown by the factor analysis results of the Early Childhood Inclusive Education Attitude Questionnaire (ECIEAQ) in this study, the Resistance, Passivity, Promotion, and Positivity dimensions of teachers’ IE attitudes can capture the ambiguities and subtleties in teachers’ IE attitudes more deeply and explore the corresponding influencing factors.

### 4.2. Factors Influencing Teachers’ IE Attitudes

Male teachers are more positive about inclusive education than female teachers, which is contrary to research in Australia and a meta-analysis of international research but consistent with results from Bangladesh [6,11,16]. However, only 4.45% of the participants in this study were male, which may have influenced the results, even though this is roughly the same as the ratio of male and female preschool teachers in China [18]. In addition, the division of labor among teachers of different genders in Asian education systems may lead to such differences. For example, women are more likely to be classroom teachers, and men are more likely to be art teachers or sports teachers, who are not in charge of any particular class but in charge of one type of curriculum for the whole preschool. Previous research has indicated that the type of teacher (classroom versus specialty) may influence the attitude of teachers about IE [16].

Compared with ICTs in rural areas, ICTs in urban areas have more positive IE attitudes, but no less negative attitudes. This is consistent with previous research findings that the rural context is a predictor of higher cognitive and behavioral dimensions of attitude [15]. Under the dual structure of urban and rural areas in China, there are differences in facilities, professionals, and funding between urban and rural preschools. Differences in resources and funding, however, should not be the cause of the differences in attitudes according to the results of this study.

Consistent with previous research, teachers who participated in IE training have more positive attitudes [3,17]. Current IE training may have a certain effect on improving teachers’ positive attitudes, but not on reducing teachers’ negative attitudes. However, Ahmmed (2009) found that IE training experience was not a predictor of IE attitude [11]. Researchers have pointed out inclusive training programs should follow international theoretical and practical approaches, as well as responding to local beliefs, assumptions, and teachers’ profiles [22]. Heterogeneous results found in different research may also be due to the heterogeneous designs of IE training programs in different countries and regions, and even in different projects, and also the cultural and local contexts of education systems. Training alone is not enough to change one’s attitude about inclusion, unless the training comprehensively addresses cultural beliefs, local traditions, and teacher profiles.

Gender, location, and IE training impact the Positivity dimension of IE attitudes, but special education financial allowance is the only categorical variable in this study that had an impact on ICTs’ Negativity dimension of attitudes. Compared to the preschool facilities and professionals examined in the preschool level rating, practical teacher welfare can improve teachers’ IE attitudes. In this study, there was no correlation between teachers’ salary levels and attitudes towards IE, but receiving a special education financial allowance significantly increased positive attitudes and reduced negative attitudes. It can be speculated that teachers are incentivized by receiving an allowance that is related to IE. A special education financial allowance can be perceived as recognition of their work in IE. Because the provision of financial incentives for inclusive education teachers is not a universally accepted practice, little research has examined this relationship; thus, this relationship and mechanism requires further exploration.

### 4.3. Interaction between Teachers’ Position and Demographics

Overall, when teachers have children with disabilities or SEN in their class, their attitudes with regard to Resistance and Promotion both improve as teachers’ attitudes became more differentiated with the implementation of IE. However, experience alone may not be adequate. MacFarlane and Wolfson (2013) found that teachers who taught children with social, emotional, and behavioral challenges were less willing to continue to have them in their classrooms [23]. Pre-service teachers have been shown to have more positive attitudes toward IE than in-service teachers [4], indicating that experience in teaching children with disabilities or SEN may not be sufficient in fostering a positive attitude about IE and may even cause a negative attitude. Likewise, Ahmmed et al. (2012) found that not experience, but successful experience in teaching students with disabilities or SEN is related to more positive attitudes towards IE [11]. These findings indicate that sufficient resources need to be provided to support teachers’ success in IE; otherwise, teachers’ attitudes towards IE may deteriorate after the implementation of IE.

Female teachers’ attitudes were more likely to be differentiated after the implementation of IE, with Resistance and Promotion attitudes improved, a phenomenon not seen among male teachers. Similarly, the attitudes of urban preschool teachers, but not rural teachers, became more differentiated after implementing IE.

Among the range of variables in this study, there are two points that may impact the difference in attitudes between ICTs and GCTs: the teacher’s age and the child-to-teacher ratio. Teachers under the age of 30 with a child-to-teacher ratio of less than 14 have an improved attitude toward Promotion after implementation of IE. Previous research found that the age of 30 or below had a similar interaction effect with prior IE training, i.e., training had more positive effect on the younger cohort [24]. Large class sizes are common in preschools in countries like China with large populations and a limited teacher population. In China, for example, class sizes can reach 30–40 children, with only two teachers. If attitudes toward IE are affected by the child-to-teacher ratio, teacher allocation may become an obstacle to the promotion of IE.

## 5. Conclusions

This study is one of the first to investigate the subtlety of teachers’ attitudes about IE. Instead of a positive–negative dichotomy, the structure of teachers’ IE attitudes is more nuanced. The influencing factors, especially on vulnerable groups whose attitudes might deteriorate after the actual implementation of IE, have been investigated. Insights into supporting the successful implementation and promotion of quality early childhood education and IE in early childhood education have been provided for teacher preparation and professional development after the passage of inclusive education legislation in China.

The variables in this study did not include teacher personality factors such as personal values of self-transcendence and openness to change, etc. [25], and student-related factors. Further investigation should be conducted to include more variables to form a more complete explanation for teachers’ attitudes. Furthermore, qualitative data should be collected through teacher interviews and observations in order to obtain in-depth insights into the attitudes of preschool teachers toward IE.

## Figures and Tables

**Table 1:** Demographic characteristics of participants.

		ICT		GCT	
		No.	%	No.	%
Gender	Male	20	4.5	14	2.2
	Female	429	95.5	624	97.8
Academic qualification	High school	5	1.1	35	5.5
	Junior college ^1^	107	23.8	213	33.4
	Bachelor	330	73.5	383	60
	Master and higher	7	1.6	7	1.1
Major	Preschool education	395	88	550	86.2
	Special education	14	3.1	2	0.3
	Others	40	8.9	86	13.4

^1^ Full-time junior colleges are usually recruited by the national unified examination for admission, with a duration of 3 years. Junior college is an important part of higher education in China and is one of the academic levels of universities.

**Table 2:** ANOVA analysis result of dimensions of ECIEAQ score.

ICTs	M	SD	F	*p*	LSD Analysis
a. Positivity	4.50	0.67	94.930	<0.001 ***	b < d < c < a ***
b. Resistance	3.60	1.15			
c. Promotion	4.30	0.75			
d. Passivity	3.80	1.00			
GCTs	M	SD	F	*p*	LSD analysis
a. Positivity	4.46	0.71	150.769	<0.001 ***	b < d < c < a ***
b. Resistance	3.44	1.10			
c. Promotion	4.14	0.77			
d. Passivity	3.80	0.97			

*** *p* < 0.001.

**Table 3:** ECIEAQ score variation across demographic groups of inclusive class teachers.

	*n*	Positivity			Resistance			Promotion			Passivity		
		M	SD	*t*	M	SD	*t*	M	SD	*t*	M	SD	*t*
Gender													
Male	20	4.75	0.42		3.55	1.16		4.45	0.56		3.87	1.09	
Female	429	4.49	0.68	2.639 **	3.61	1.15	−0.214	4.29	0.76	0.921	3.80	0.99	0.305
Preschool location													
Urban	393	4.53	0.65		3.62	1.15		4.33	0.74		3.82	1.00	
Rural	56	4.27	0.82	7.792 ***	3.47	1.13	0.867	4.11	0.80	4.163 *	3.65	0.97	1.482
Inclusive training													
No	121	4.28	0.75		3.73	1.04		4.00	0.78		3.87	0.96	
Yes	328	4.58	0.62	18.206 ***	3.56	1.18	2.114	4.41	0.71	26.787 ***	3.77	1.01	0.824
WEFA													
No	349	4.46	0.68		3.68	1.09		4.24	0.77		3.86	0.97	
Yes	100	4.66	0.62	7.103 ***	3.33	1.28	7.262 ***	4.50	0.65	9.164 ***	3.61	1.07	4.907 **

* *p*< 0.05, ** *p* < 0.01, *** *p* < 0.001; Note. WEFA—special education financial allowance.

**Table 4:** Comparison of ICTs and GCTs: Subgroups where differences exist.

		*n*	Positivity			Resistance			Promotion			Passivity		
			M	SD	*t*	M	SD	*t*	M	SD	*t*	M	SD	*t*
Full sample	GCTs	638	4.46	0.71		3.44	1.10		4.14	0.77		3.80	0.97	
	ICTs	449	4.50	0.67	1.086	3.60	1.15	5.710 *	4.30	0.75	12.901 **	3.80	1.00	0.001
Gender: Female	GCTs	624	4.45	0.72		3.45	1.10		4.13	0.77		3.80	0.97	
	ICTs	429	4.49	0.68	0.979	3.61	1.15	2.277 *	4.29	0.76	3.412 **	3.80	0.99	0.006
Age ≤ 30	GCTs	364	4.47	0.68		3.42	1.12		4.12	0.74		3.80	0.94	
	ICTs	284	4.56	0.67	2.523	3.56	1.17	2.285	4.34	0.72	14.077 ***	3.81	1.02	0.023
Age >31	GCTs	274	4.43	0.75		3.46	1.07		4.15	0.80		3.80	1.01	
	ICTs	165	4.40	0.68	0.190	3.68	1.09	4.264 *	4.22	0.80	0.870	3.79	0.96	0.020
Child-to-teacher ratio ≤1	GCTs	345	4.50	0.69		3.40	1.15		4.14	0.81		3.78	1.01	
	ICTs	283	4.50	0.69	0.002	3.54	1.13	2.559	4.29	0.77	5.557 *	3.78	0.96	0.001
Child-to-teacher ratio > 14	GCTs	293	4.41	0.73		3.49	1.04		4.13	0.72		3.82	0.92	
	ICTs	166	4.51	0.64	2.303	3.70	1.17	4.237 *	4.32	0.72	6.944 **	3.84	1.06	0.902
Urban preschool	GCTs	527	4.45	0.72		3.44	1.11		4.14	0.77		3.77	0.99	
	ICTs	393	4.53	0.65	3.119	3.62	1.15	6.200 *	4.33	0.74	12.899 ***	3.82	1.00	0.533

* *p* < 0.05, ** *p* < 0.01, *** *p* < 0.001.

## Data Availability

Data are unavailable due to privacy restrictions.
